# Host miR-146a-3p Facilitates Replication of Infectious Hematopoietic Necrosis Virus by Targeting WNT3a and CCND1

**DOI:** 10.3390/vetsci11050204

**Published:** 2024-05-08

**Authors:** Jingwen Huang, Shihao Zheng, Qiuji Li, Hongying Zhao, Xinyue Zhou, Yutong Yang, Wenlong Zhang, Yongsheng Cao

**Affiliations:** 1 College of Veterinary Medicine, Northeast Agricultural University, Changjiang Street NO.600, Harbin 150030, China; 2 Northeastern Science Inspection Station, China Ministry of Agriculture Key Laboratory of Animal Pathogen Biology, Harbin 150069, China

**Keywords:** infectious hematopoietic necrosis virus, miRNA-146a-3p, interferon, viral replication, WNT3a, CCND1

## Abstract

**Simple Summary:**

Infectious hematopoietic necrosis virus (IHNV) is listed in the World Organization for Animal Health (WOAH) for its serious infection in salmonid fish. MicroRNAs (miRNA) were important regulators in the immune responses during viral infections. However, the roles of miRNAs during IHNV infection are rarely known. Our previous work found that IHNV infection upregulated the expression of miRNA146a-3p. The present study is intended to detail the molecular mechanism underlying the role of miRNA146a-3p in IHNV infection. This will provide new insight into IHNV pathopoiesis and might benefit the development of antiviral strategies against IHNV.

**Abstract:**

Infectious hematopoietic necrosis virus (IHNV) is a serious pathogen that causes great economic loss to the salmon and trout industry. Previous studies showed that IHNV alters the expression patterns of splenic microRNAs (miRNAs) in rainbow trout. Among the differentially expressed miRNAs, miRNA146a-3p was upregulated by IHNV. However, it is unclear how IHNV utilizes miRNA146a-3p to escape the immune response or promote viral replication. The present study suggested that one multiplicity of infection (MOI) of IHNV induced the most significant miR-146a-3p expression at 1 day post infection (dpi). The upregulation of miR-146a-3p by IHNV was due to viral N, P, M, and G proteins and relied on the interferon (IFN) signaling pathway. Further investigation revealed that Wingless-type MMTV integration site family 3a (WNT3a) and G1/S-specific cyclin-D1-like (CCND1) are the target genes of miRNA-146a-3p. The regulation of IHNV infection by miRNA-146a-3p is dependent on WNT3a and CCND1. MiRNA-146a-3p was required for the downregulation of WNT3a and CCND1 by IHNV. Moreover, we also found that WNT3a and CCND1 are novel proteins that induce the type-I IFN response in RTG-2 cells, and both of them could inhibit the replication of IHNV. Therefore, IHNV-induced upregulation of miRNA-146a-3p promotes early viral replication by suppressing the type-I IFN response by targeting WNT3a and CCND1. This work not only reveals the molecular mechanism of miRNA-146a-3p during IHNV infection but also provides new antiviral targets for IHNV.

## 1. Introduction

The rainbow trout (*Oncorhynchus mykiss*) is a *Salmonidae* fish living in the cold water. The global aquaculture production of rainbow trout was 959,600 tons in 2020 [1]. Unfortunately, infectious diseases cause problems in farmed trout, resulting in significant economic losses. Among these, the infectious hematopoietic necrosis virus (IHNV) is a serious and economically important viral pathogen. When mass mortality occurs in hatcheries, the overall population can be infected, with 90% mortality [2].

IHNV is a single-stranded negative-sense RNA virus of the *Novirhabdovirus* genus within the *Rhabdoviridae* family. The complete genome of IHNV comprises approximately 11,000 nucleotides and contains six genes encoding the nucleoprotein (N), polymerase-associated phosphoprotein (P), matrix protein (M), glycoprotein (G), non-structural protein (NV), and RNA-dependent RNA polymerase (L), respectively. In addition to rainbow trout, various salmonids, including Atlantic salmon (*Salmo salar*), Chinook salmon (*Oncorhynchus tshawytscha*) andMasou salmon (*Oncorhynchus masou*), are naturally sensitive to IHNV [3]. This virus mainly affects fry and fingerlings. However, it has been reported that adult fish can also be infected by IHNV. Viral particles have also been detected in survivors [4]. This brings new challenges to IHNV infection control.

Interferons (IFN) play important roles in the host defense during viral infection. In addition to antiviral protection, interferon can also enhance adaptive immunity [5]. In our previous work, recombinant interferon proteins from rainbow trout were shown to elicit a significant antiviral response and provide efficient protection against IHNV [6]. The early protection provided by the vaccine is closely associated with the immediate IFN response [7]. However, IHNV can escape the IFN system through multiple strategies. For example, the IHNV N protein targets MITA to inhibit IFN1 production [8]. The IHNV NV protein recruits the cellular PPM1Bb protein phosphatase to antagonize RIG-I-mediated IFN induction [9]. The IHNV matrix protein inhibits the activity of the IFN promoter and induces apoptosis in cell cultures [10]. IHNV P and G proteins have also been shown to possess slight IFN inhibitory activities [11].

The interaction between viruses and their hosts has been the focus of recent years. Based on these studies, the pathogenic mechanisms have been better classified, thus benefiting the development of antiviral drugs [12]. MicroRNA (miRNA) is a form of small noncoding RNA that plays a crucial role in immune regulation [13]. The interaction between miRNAs and IFN during infection has been studied in multiple aquatic viruses [14]. MiR-122 reduces MAVS expression to suppress MAVS-mediated antiviral responses and benefits *Siniperca chuatsi* rhabdovirus (SCRV) replication [15]. MiR-217 is upregulated during SCRV infection and inhibits NF-κB- and IRF3-driven immune responses by targeting TAK1 [16]. Megalocytivirus induces pol-miR-731, which negatively regulates virus-induced type I IFN response [17]. In contrast, miR-155 regulates the AMPK-MAVS-IFN pathway to inhibit cyprinid herpesvirus 3 replication [18]. MiR-214 targets the host adenosine 5′-monophosphate-activated protein kinase to inhibit snakehead vesiculovirus replication by the increased expression of IFN-α [19].

We previously found that IHNV infection induced significant expressions of miRNA-146a-3p, which could inhibit the IFN-related genes and promote viral replication [20]. In this study, miRNA-146a-3p was identified as an early regulator of IHNV infection. The induction of miRNA-146a-3p by IHNV was due to viral N, P, M, and G proteins via the Retinoic acid-inducible gene I (RIG-I)/NF-κB axis and IFN-mediated signaling. Furthermore, Wingless-type MMTV integration site family 3a (WNT3a) and G1/S-specific cyclin-D1-like (CCND1) were proved to be the target genes of miRNA-146a-3p. The downregulation of WNT3a and CCND1 during early IHNV infection was dependent on miRNA-146a-3p. Finally, we determined that miRNA-146a-3p promoted IHNV infection by inhibiting the IFN response via reducing WNT3a and CCND1 expression. This study details how IHNV utilizes host miR-146a-3p to evade the type I interferon system. This may help to better understand the molecular mechanism of IHNV pathogenicity and thereby provide more information for the design of anti-IHNV agents.

## 2. Materials and Methods

### 2.1. Cells and Virus

RTG-2 cells, a line of fibroblasts from rainbow trout cells (ATCC CCL-55), were grown at 20 °C in Eagle’s minimal essential medium (EMEM) (HyClone, Logan, UT, USA) supplemented with 10% fetal bovine serum (Wisent, Saint-Jean-Baptiste, QC, Canada), penicillin (100 U/mL), and streptomycin (0.1 mg/mL) solution (Biosharp, Hefei, China). IHNV was isolated from diseased rainbow trout fry and stored at −80 °C.

### 2.2. Reagents and Antibodies

Pyrrolidinedithiocarbamate ammonium (PDTC, HY-18738) and carbonyl cyanide 3-chlorophenylhydrazone (CCCP, HY-100941) were purchased from MedChemExpress. Recombinant iIFN1a protein was generated in our previous study and stored at −80 °C [6]. Rabbit anti-WNT3a polyclonal antibody (D122111), rabbit anti-CCND1 polyclonal antibody (D160236), and HRP-conjugated goat anti-rabbit IgG were purchased from Sangon Biotech (Shanghai, China). Rabbit monoclonal antibodies against beta-tubulin were obtained from Abcam (Cambridge, UK; EPR16774).

### 2.3. Virus Infection and Titer Determination

To analyze whether the expression of miR-146a-3p depended on the multiplicity of infection (MOI) of IHNV, RTG-2 cells were incubated with 10, 1, or 0.1 MOI of IHNV for 3 h at 15 °C. The medium was replaced with EMEM containing 2% FBS. The 1 MOI of IHNV was used to infect RTG-2 cells that had been transfected with plasmids, miRNA mimics, miRNA inhibitors, or siRNA for 24 h. At different time points after virus infection, the cells were collected for the detection of the expression of miRNAs, viral genes, or immune genes by quantitative reverse transcription-PCR (qRT-PCR). The supernatants were collected to measure the 50% tissue culture infective dose (TCID50) using the Reed and Muench method.

### 2.4. miRNA Mimics and Inhibitors

The miR-146a-3p mimic (double-stranded RNA oligonucleotides), inhibitor (single-stranded RNA oligonucleotides), and negative control (NC) mimics or inhibitors were designed and synthesized by GenePharma (Shanghai, China). The overexpression or inhibition of miR-146a-3p in RTG-2 cells was accomplished via transfection with miRNA mimics or inhibitors by following our previous methods [20].

### 2.5. Plasmid Construction

Based on the reference sequence of WNT3a (XM_021614260.2), the DNA fragment corresponding to the open frame (ORF) of WNT3a was synthesized by Comate Bioscience Co., Ltd. (Changchun, China). The gene was then cloned into the pcDNA3.1 vector to construct the expression plasmid pWNT3a. The expression plasmid pCCND1 was generated by amplifying the ORF of the CCND1 gene (XM_036959225.1) from the splenic RNAs of rainbow trout and cloning it into the pcDNA3.1. The luciferase reporter plasmid pmirGLO-WNT3a or pmirGLO-CCND1 was generated by amplifying the miR-146a-3p target sequence (~200 nt) in the 3′ UTR of WNT3a or CCND1, respectively, and cloned into pmirGLO. The luciferase reporter plasmids pmirGLO-WNT3a-MUT or pmirGLO-CCND1-MUT containing the mutated target sequence of miR-146a-3p were constructed using PCR-mediated mutations in the corresponding plasmid. A sequence of *GCCACC***ATG**GCTTGGAGCCACCCGCAGTTCGAAAAGGGTGGCGGGAGTGGCGGGGGTAGTGGCGGTAGCGCATGGAGTCATCCCCAGTTTGAGAAG corresponding to the Kozak sequence (italic), initiation codon (bold), and the gene encoding the twin-strep tag [21] was synthesized and cloned into pcDNA3.1 at *Nhe* I and *Hind* III sites by Comate Bioscience Co., Ltd. (Changchun, China). Based on this vector, the plasmids pN, pP, pM, pG, pNV, pL-1 (1–673 aa of L protein), pL-2 (674–1380 aa of L protein), and pL-3 (1381-1987 aa of L protein) were constructed by routine molecular cloning using the IHNV genomic RNA as a template. The plasmids were constructed using the primers listed in Table 1.

### 2.6. Cell Transfection

RTG-2 cells were cultured until they reached 80% confluence in 6-well plates. The mimic, inhibitor, or plasmid was mixed with GP-transfect-Mate (GenePharma, Shanghai, China) in an Opti-MEM medium (Invitrogen, Carlsbad, CA USA) at a volume of 100 μL for 20 min. After 6 h incubation, the transfection mixture was replaced with 2 mL fresh EMEM.

### 2.7. RNA Interference

Small RNAs (siRNAs) targeting IFN receptor (forward, 5′-CUCGCUACGACUACUACAATT-3′; reverse, 5′-UUGUAGUAGUCGUAGCGAGTT-3′), STAT1 (forward, 5′-GCGAAGAGGAGAUUGUCAUTT-3′; reverse, 5′-AUGACAAUCUCCUCUUCCTT-3′), WNT3a (forward, 5′-GGAGUCAGCAUUCGUUCAUTT-3′; reverse, 5′-AUGAACGAAUGCUGACUCCTT-3′), CCND1 (forward, 5′-GCAGAGAAGUUGUGCAUAUTT; reverse, 5′-AUAUGCACAACUCUCUGCTT-3′) or NC siRNA (forward, 5′-UUCUCCGAACGUGUCACGUTT-3′; reverse, 5′-ACGUGACACGUUCGGAGAATT-3′) were designed and synthesized by GenePharma (Shanghai, China). The siRNAs or NC siRNA was transfected into RTG-2 cells at a concentration of 50 nM using GP-transfect-Mate (GenePharma, Shanghai, China). Twenty-four hours later, the cells were infected with 1 MOI IHNV. After 24 h, cells were collected for RNA isolation and real-time PCR detection.

### 2.8. Quantitative Reverse Transcription-PCR (qRT-PCR)

Total RNAs were isolated from the cells using a commercial kit (Axgen, Corning, NY, USA). The expression of miR-146a-3p was analyzed using an Applied Biosystems 7500 system and the All-in-One™ miRNA qRT-PCR Detection Kit (GeneCopoeia, Rockville, MD, USA). For the detection of viral gene N, target genes, and IFN-related genes, reverse transcription was performed using ReverTra Ace^®^ qPCR RT Master Mix with gDNA Remover kit (TOYOBO, Osaka, Japan), followed by qRT-PCR detection with NovoStart^®^ SYBR qPCR SuperMix Plus kit (Novoprotein, Suzhou, China). The miRNA or gene data were normalized to the level of 18s rRNA or β-actin, respectively. The primers used for real-time PCR are listed in Table 1. The experiments were performed in triplicate and the data were analyzed using the 2^−ΔΔCT^ method.

### 2.9. Luciferase Reporter Assays

HEK293T cells were cultured in 6-well plates until they reached 80% confluence. The cells were transfected with miR-146a-3p, together with pmirGLO-WNT3a, or pmirGLO-CCND1 using Lipofectamine 2000 transfection reagent (Invitrogen). The NC mimic, pmirGLO-WNT3a-MUT, or pmirGLO-CCND1-MUT was transfected into cells as a control. After 24 h, the cells were washed with PBS and lysed to measure Renilla and firefly luciferase activity using the Dual-Luciferase^®^ Reporter Assay System (Promega, Madison, WI, USA). The relative activity of firefly luciferase was normalized to that of Renilla luciferase.

### 2.10. Immunoblot

Cells were lysed using Cell Lysis Buffer for Western & IP (Genstar, Beijing, China) containing Protease Inhibitor Cocktail (Sigma, Madison, WI, USA). For Western blotting experiments, cellular proteins were separated by SDS-PAGE and transferred onto a nitrocellulose membrane. The primary antibodies were anti-WNT3a, anti-CCND1, and anti-β tubulin. After incubation with HRP-conjugated anti-rabbit/mouse IgG, immunolabeled bands were visualized using an ECL kit (P0018S, Beyotime, Shanghai, China).

### 2.11. Statistical Analysis

Each determination was performed in triplicate (mean ± SD). Comparisons between different groups were statistically analyzed by Student’s t-test using Graphpad Prism 5.0 (GraphPad Software, La Jolla, CA, USA). The differences were considered statistically significant (* *p* < 0.05; ** *p* < 0.01).

## 3. Results

### 3.1. IHNV Infection Upregulated the Expression of miR-146a-3p

**Figure 1:**
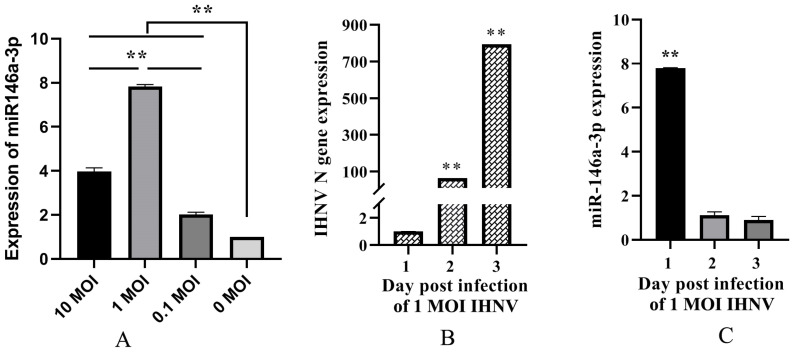
IHNV infection upregulated the expression of miRNA-146a-3p in RTG-2 cells. (**A**) RTG-2 cells were infected with IHNV at an MOI of 10, 1, and 0.1, and the level of miRNA-146a-3p at 1 day post infection (dpi) was measured by qRT-PCR. RTG-2 cells were infected with IHNV at an MOI of 1, and the levels of IHNV N gene (**B**) and miRNA-146a-3p (**C**) at 1, 2, and 3 dpi were measured by qRT-PCR. The ** indicates statistically significant differences (** *p* < 0.01).

To determine the relationship between IHNV infection and miR-146a-3p, RTG-2 cells were infected with IHNV at 10, 1, and 0.1 MOI. As a result, IHNV infection induced the upregulation of miR-146a-3p. Compared with 10 and 0.1 MOI, 1 MOI of IHNV caused the most significant miR-146a-3p expression (Figure 1A). Therefore, 1 MOI of IHNV was used to infect the cells in the following experiment. With the replication of IHNV (Figure 1B), significant upregulation of miR-146a-3p was observed only at 1 day post infection (dpi) (Figure 1C). This indicates that miR-146a-3p might be functional in the early stages of IHNV infection.

### 3.2. The Induction of miR-146a-3p Was Associated with IHNV Inducing IFN Response

**Figure 2:**
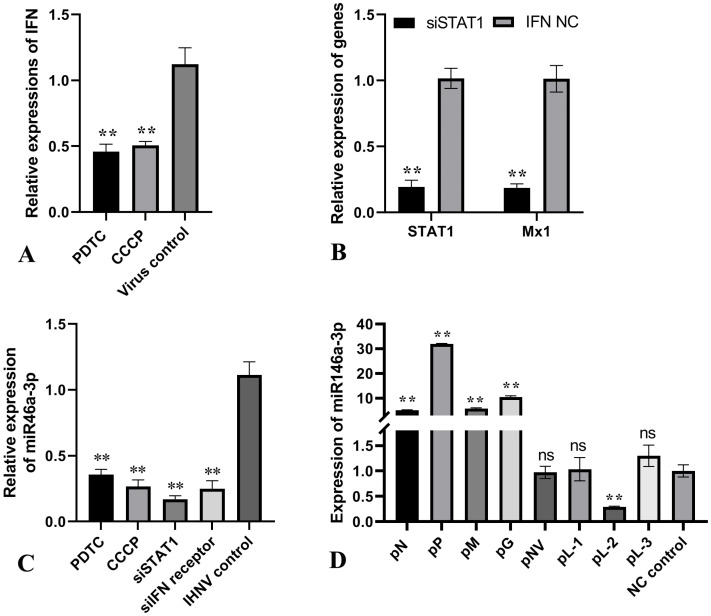
IHNV-induced miRNA-146a-3p expression was associated with IFN signaling. (**A**) RTG-2 cells were incubated with 10 μM of PDTC or CCCP for 24 h, followed by 1 MOI of IHNV infection. The level of the IFN gene was measured by qRT-PCR and compared with that of the IHNV-infected mock cells. (**B**) RTG-2 cells were transfected with 50 nM of STAT1-targeted siRNA, followed by incubation with recombinant iIFN1a protein at a concentration of 500 IU/mL. The levels of the STAT1 gene and Mx1 gene were measured by qRT-PCR and compared with that of the recombinant iIFN1a protein incubated mock cells. (**C**) RTG-2 cells were treated with PDTC, CCCP, IFN receptor-targeted siRNA, or STAT1-targeted siRNA, followed by 1 MOI of IHNV infection. The level of miRNA-146a-3p was measured by qRT-PCR and compared with that of the IHNV-infected mock cells. (**D**) RTG-2 cells were transfected with eight plasmids expressing each IHNV protein (among these, L protein was truncated to three segments), respectively. Twenty-four hours later, the expression of miRNA-146a-3p was measured by qRT-PCR. The ** indicates statistically significant differences (** *p* < 0.01).

Previous functional studies on miRNA-146a in mammals have shown that miR-146a is a feedback regulator of the type I IFN response [22]. To investigate whether IHNV-induced miR-146a-3p was also IFN-dependent, chemical inhibitors and small interfering RNAs were used to establish an IFN-less responsive model. PDTC inhibits the activation of NF-κB [23], whereas CCCP suppresses STING-mediated IFN-β production [24]. Figure 2A showed that pretreatment with PDTC or CCCP was effective in inhibiting IFN signaling in RTG-2 cells upon IHNV infection. Meanwhile, the expression of Mx1 was suppressed when endogenous STAT1 expression was inhibited by siRNA (Figure 2B). When the cells were infected with IHNV, the expression of miR-146a-3p in the IFN-less responsive cells was significantly lower than that in the mock cells (Figure 2C). Based on the functional properties of the above drugs and siRNAs, we proposed that IHNV-induced miR-146a-3p was dependent on the RIG-I/NF-κB pathway and IFN-mediated signaling. To evaluate whether the expression of miR-146a-3p depended on viral proteins, eight plasmids expressing each IHNV protein (among these, the L protein was truncated to three segments) were constructed and transfected into cells. MiR-146a-3p was found to be significantly upregulated in IHNV N, P, M, and G protein-transfected cells. However, transfection with pL-2 repressed miR-146a-3p expression (Figure 2D).

### 3.3. Identification of the Potential Target Genes of miR-146a-3p

**Figure 3:**
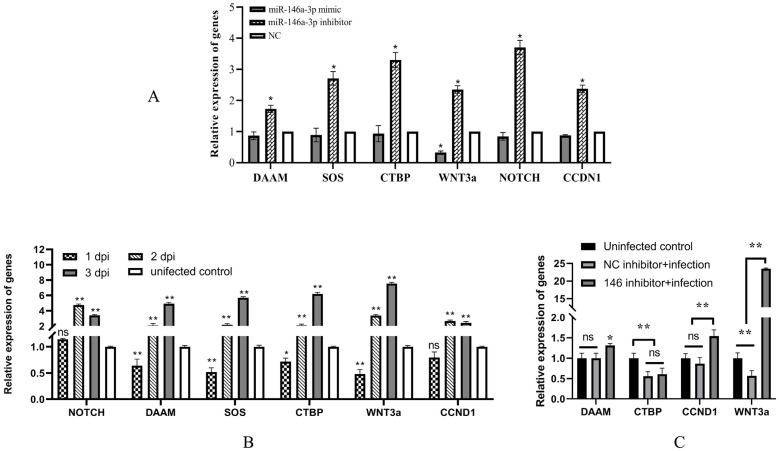
Identification and characterization of the potential target genes of miRNA-146a-3p. (**A**) Validation of the potential target genes of miRNA-146a-3p by qRT-PCR. miRNA-146a-3p mimic or inhibitor was transfected into RTG-2. Twenty-four hours later, the levels of DAAM, SOS, CTBP, WNT3a, NOTCH, and CCND1 were measured by qRT-PCR. (**B**) Analysis of the potential target genes of miRNA-146a-3p in responding to IHNV infection. RTG-2 cells were infected with 1 MOI of IHNV or untreated, followed by the detection of genes using qRT-PCR. (**C**) Downregulations of the potential target genes by IHNV were dependent on miRNA-146a-3p. RTG-2 cells were transfected with miRNA-146a-3p inhibitor and NC inhibitor, followed by 1 MOI of IHNV infection. The * and ** indicate statistically significant differences (* *p* < 0.05; ** *p* < 0.01).

To determine the target genes, miR-146a-3p mimic or NC mimic was transfected into RTG-2 cells, followed by high-throughput sequencing. A total of 497 genes were found to be differentially expressed upon the overexpression of miR-146a-3p (255 downregulated genes and 242 upregulated genes). Among these, six downregulated target genes that were associated with immune response or signal transduction were selected for further validation by qRT-PCR (Table 2). Consistent with the high-throughput sequencing results, the overexpression of miR-146a-3p slightly downregulated the expression of the neurogenic locus notch homolog protein 2-like (NOTCH), disheveled-associated activator of morphogenesis 1 (DAAM), son of sevenless homolog 1 isoform X2 (SOS), C-terminal-binding protein 1 isoform X3 (CTBP), and CCND1, but significantly suppressed the expression of WNT3a. In contrast, the inhibition of miR-146a-3p expression significantly enhanced the expression of these six genes (Figure 3A).

### 3.4. IHNV Downregulates the Expression of Potential Target Genes at Early Time after Infection

To preliminarily analyze the relationship between the potential target genes of miR-146a-3p and IHNV infection, their expression at 1, 2, and 3 dpi was detected and compared with that of the uninfected control. The results showed that except NOTCH and CCND1, the expressions of DAAM, SOS, CTBP, and WNT3a were all downregulated at 1 dpi. However, all six potential target genes were upregulated at 2 and 3 dpi (Figure 3B). Figure 1 shows that the significantly upregulated expression of miR-146a-3p was observed only at 1 dpi. Therefore, the expression of miR-146a-3p and DAAM, SOS, CTBP, and WNT3a were in accordance with the regulation of miRNAs on target genes.

### 3.5. miR-146a-3p Participates in the Repression of Potential Target Genes by IHNV

To investigate whether miR-146a-3p plays a crucial role in the downregulation of potential target genes by IHNV, cells were transfected with miR-146a-3p inhibitor or NC inhibitor, followed by IHNV infection. The results indicated that IHNV infection caused the downregulation of DAAM, CTBP, CCND1, and WNT3a. Suppression of miR-146a-3p upregulated DAAM, CCND1, and WNT3a expression levels instead (Figure 3C). This suggests that IHNV represses DAAM, CCND1, and WNT3a via miR-146a-3p.

### 3.6. WNT3a and CCND1 Are the Target Genes of miR-146a-3p

**Figure 4:**
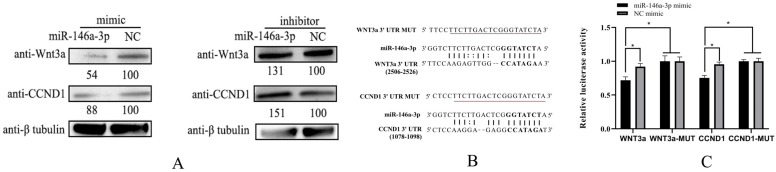
WNT3a and CCND1 were targeted by miRNA-146a-3p. (**A**) Western blotting analysis. RTG-2 cells were transfected with miRNA-146a-3p mimic or NC mimic and miRNA-146a-3p inhibitor or NC inhibitor for 24 h. Then, the cells were lysed and the target protein bands were detected using the corresponding antibody. (**B**) Alignment of miR-146a-3p and the putative target sequences in the 3′ UTR of WNT3a and CCND1. (**C**) HEK293T cells were transfected with pmirGLO-WNT3a, pmirGLO-WNT3a-MUT, pmirGLO-CCND1, or pmirGLO-CCND1-MUT, together with miR-146a-3p mimic or NC mimic, for 24 h as indicated. Luciferase activity was measured and normalized to Renilla luciferase activity. The * indicates statistically significant differences (* *p* < 0.05).

To further determine the effects of miR-146a-3p on the expression of CCND1 and WNT3a, the cells were transfected with miR-146a-3p mimic, miR-146a-3p inhibitor, NC mimic, or NC inhibitor. CCND1 and WNT3a proteins were detected by Western blotting. The overexpression of miR-146a-3p reduced cellular CCND1 and WNT3a protein expression. In contrast, the repression of miR-146a-3p-enhanced cellular CCND1 and WNT3a protein expression (Figure 4A). To confirm the miR-146a-3p-target interactions, Mirnada software was used to identify the putative binding sites of miR-146a-3p at the 3′ UTR of CCND1 and WNT3a (Figure 4B). Based on this, dual-luciferase reporter plasmids containing the wild-type sequence of the 3′ UTR of CCND1 and WNT3a, or the corresponding mutant reporter plasmids, were generated. The reporter plasmid was transfected into HEK 293T cells together with miR-146a-3p mimic or NC mimic. The luciferase activity in cells co-transfected with the miR-146a-3p mimic and reporter plasmid containing the wild-type sequence of the 3′ UTR of the gene was significantly lower than that in cells co-transfected with the NC mimic and reporter plasmid. However, this effect was not observed in the mutant reporter plasmid transfection group (Figure 4C). Collectively, WNT3a and CCND1 are targets of miR-146a-3p (Supplementary Materials Figure S1).

### 3.7. The Roles of miR-146a-3p in IHNV Infection Were Dependent on WNT3a and CCND1

**Figure 5:**
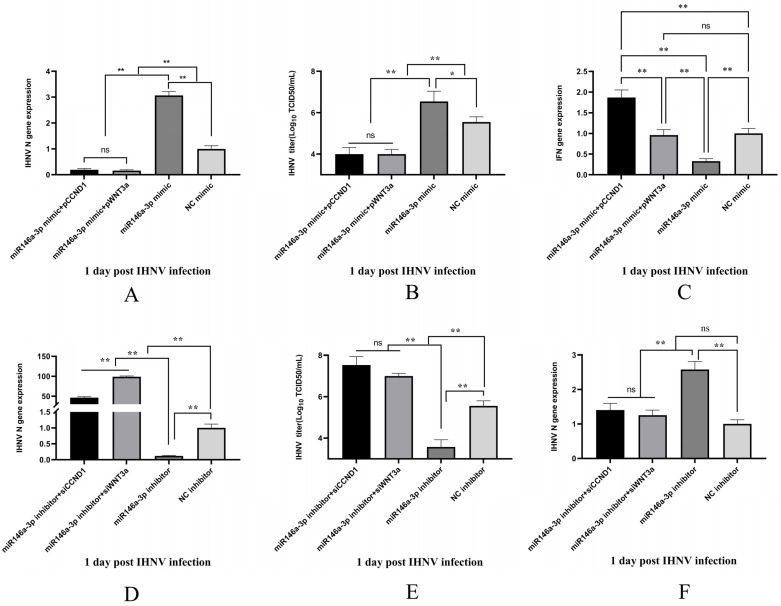
miR-146a-3p regulation of IHNV infection was in the WNT3a and CCND1-dependent manner. RTG-2 cells were transfected with miR-146a-3p mimic or NC mimic, or cotransfected with miRNA-146a-3p mimic and pCCDN1/pWNT3a, or cotransfected with miRNA-146a-3p inhibitor and siCCDN1/siWNT3a. Twenty-four hours later, the expressions of the viral gene and IFN gene were detected by qRT-PCR. The amount of virus in the supernatant was measured using the TCID_50_ assay. The * and ** indicate statistically significant differences (* *p* < 0.05; ** *p* < 0.01).

Our previous work shows that miRNA-146a-3p inhibits IFN-related gene expression and promotes viral replication. To explore whether the roles of miR-146a-3p were in a WNT3a- or CCND1-dependent manner, several experimental groups were set as follows: miRNA-146a-3p mimic transfection group, miRNA-146a-3p mimic and pCCDN1/pWNT3a co-transfection group, miRNA-146a-3p inhibitor transfection group, miRNA-146a-3p inhibitor and siCCDN1/siWNT3a co-transfection, NC mimic transfection, and NC inhibitor groups. Transfected cells were infected with IHNV 24 h later. Viral replication and IFN responses were determined. As shown in Figure 5, the enhancement of viral replication by the miRNA-146a-3p mimic was reversed to inhibition of viral replication via supplementation with pCCND1 or pWNT3a. IFN gene expression was restored or even upregulated by the addition of pWNT3a or pCCND1. In contrast, the inhibition of viral replication by miRNA-146a-3p inhibitor was reversed to enhance viral replication by siRNA-mediated CCND1 or WNT3a knockdown. The upregulation of IFN gene expression was attenuated by siRNA-mediated CCND1 or WNT3a knockdown. Overall, it is clearly shown that WNT3a and CCND1 are involved in the miR-146a-3p-mediated regulation of IHNV infection.

### 3.8. WNT3a and CCND1 Inhibited IHNV Replication

**Figure 6:**
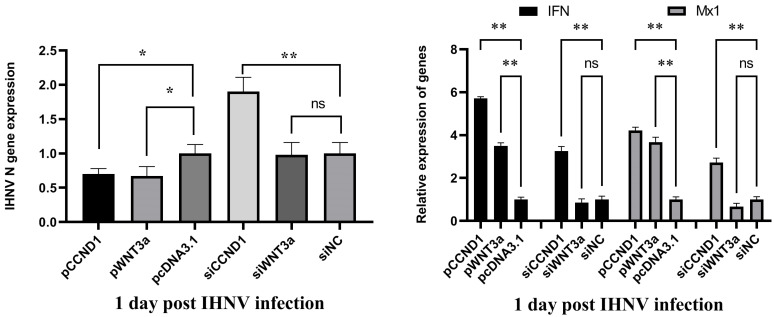
WNT3a and CCND1 inhibited IHNV replication. RTG-2 cells were transfected with pWNT3a, pCCND1, siWNT3a, and siCCND1, respectively. Twenty-four hours later, the cells were infected with IHNV. Another 24 h post infection, the expressions of viral gene and IFN-related genes were analyzed by qRT-PCR. The * and ** indicate statistically significant differences (* *p* < 0.05; ** *p* < 0.01).

To explore the roles of WNT3a and CCND1 in the regulation of IHNV infection, pWNT3a, pCCND1, siWNT3a, and siCCND1 were transfected into RTG-2 cells, respectively. Twenty-four hours later, cells were infected with IHNV. Another 24 h post infection, the expression of viral and IFN-related genes was determined. The results showed that the overexpression of WNT3a and CCND1 inhibited IHNV replication and enhanced IFN responses. The suppression of WNT3a restored viral replications, whereas the suppression of CCND1 enhanced viral replications. The knockdown of WNT3a restored IFN responses, but the knockdown of CCND1 enhanced IFN responses (Figure 6).

## 4. Discussion

MicroRNAs (miRNAs) are important post-transcriptional regulators in animals. Cellular miRNA expression profiles are altered during viral infection. Differentially expressed miRNAs participate in the regulation of viral replication and numerous signaling pathways [25]. Studies on miRNAs during viral infection not only provide new information about the interaction between the virus and host but also benefit the development of therapeutic approaches against viral infection [26].

Recently, various fish miRNAs have been identified and proven to be functional in different aquatic viral infections [14]. Our previous work showed that miR-146a-3p was associated with IHNV infection. The miR-146a precursor is cleaved by cytoplasmic Dicer ribonuclease to generate mature miRNA (miR-146a-5p) and antisense miRNA (miR-146a-3p). miR-146a-5p is a well-known dominant negative regulator of the IFN pathway [27]. However, our previous study found that IHNV did not induce changes in the miR-146a-5p expression but upregulated the expression of miR-146a-3p. During viral infection, miR-146a-5p suppresses the production of type-I interferon by targeting not only IRAK2, TRAF6, and IRAK1 [22] but also STAT-1 and IRF-5 [28]. Although miR-146a-3p also affects the type-I interferon response, the above five signaling components/molecules are not predicted as the target genes of miR-146a-3p in rainbow trout.

To obtain more information about how miR-146a-3p is induced by IHNV, RTG-2 cells were infected with IHNV at three different MOIs. Compared with 10 and 0.1 MOI, 1 MOI of IHNV induced the most significant expression of miR-146a-3p. Additionally, 1 MOI of IHNV caused the upregulation of miR-146a-3p only at 1 dpi (Figure 1). miRNAs are known to be part of the antiviral response during the early phases of viral infections [29]. We previously found that miR-146a-3p suppresses the type-I interferon response. Hence, it is speculated that IHNV might take advantage of cellular miR-146a-3p to initiate efficient infection.

Type I interferon production caused by endosomal TLR activation during hepatitis C virus (HCV) infection rapidly modulates the expression of numerous host miRNAs in infected hepatocytes [30]. Vesicular stomatitis virus (VSV) (belonging to *Rhabdoviridae* family) induces miR-155 and miR-223 via the RIG-I/JNK/NF-κB pathway [31]. Considering that innate immune sensors have also been proven to detect viral compounds at the early stage of IHNV infection [32], the relationship between the initiation of anti-IHNV signaling cascades and the expression of miR-146a-3p was analyzed. The cells were treated with siRNAs and chemical inhibitors, followed by infection with IHNV. The expression of miR-146a-3p was compared to that in untreated cells upon IHNV infection (Figure 2). It can be concluded that in an infection by IHNV, miR-146a-3p expression was induced in RTG-2 cells through a RIG-I/NF-κB-dependent pathway and was also associated with the IFN signaling pathway.

miRNAs are involved in viral infection by regulating the immune-related genes or directly targeting the viral genome [33]. The target genes were predicted based on the sequence of miR-146a-3p and seed complementarity to the 3′-UTR of the IHNV genome or host coding genes. However, no miRNA-binding sites were found in the IHNV genes. Thus, the function of miR-146a-3p is proposed to be accomplished by regulating host genes. However, information regarding the miRNAs of rainbow trout and their targets is still limited. Based on the differentially expressed genes in the miR-146a-3p over-expressed RTG-2 cells, target genes were predicted using miRanda software [34]. The expression of putative target genes was further validated in the miR-146a-3p mimic- or inhibitor-transfected cells. The suppression of miR-146a-3p enhances the expression of putative target genes. Corresponding to the significant upregulation of miR-146a-3p, the putative target genes (DAAM, SOS, CTBP, WNT3a, and CCND1) were only downregulated at 1 day post IHNV infection. Additionally, the inhibition of miR-146a-3p reversed the suppression of CCND1 and WNT3a by IHNV infection. Finally, using classical approaches for miRNA-target identification, CCND1 and WNT3a were confirmed to be the target genes of miR-146a-3p.

Similar to other teleost miRNAs, including Miiuy croaker miR-122 [15] and miiuy croaker miR-217 [16], Japanese flounder miR-731 [17], common carp miR-155 [18], and snakehead miR-214 [19], trout miR-146a-3p is also a putative type I interferon regulator. However, both prediction and experimental evidence indicated that the target genes of miR-146a-3p were not IFN-signaling components or molecules. To confirm whether CCND1 and WNT3a were involved in the regulation of IHNV infection by miR-146a-3p, CCND1 and WNT3a were added back or knocked down in miR-146a-3p-treated cells. In fact, CCND1 and WNT3a negatively affected the action of miR-146a-3p in IHNV-infected cells (Figure 5).

Both WNT3a and CCND1 are key components of the WNT signaling pathway. WNT expression and signaling are central regulators of embryonic development and tissue homeostasis. However, the WNT signaling pathway has recently been shown to shape antiviral responses upon infection [35]. The roles of WNT expression, signaling, and its function in viral infection vary among viruses. For example, Epstein–Barr virus infection upregulates the expression of WNT5A and activates WNT signaling [36]. Porcine circovirus-like virus P1 utilizes viral protein VP1 to inhibit Wnt/β-catenin signaling [37]. In turn, the active WNT signaling pathway was able to suppress the inflammatory response and promote the replication of porcine reproductive and respiratory syndrome viruses [38]. When the Wnt/β-catenin pathway was activated by WNT3a, influenza virus mRNA and virus production were enhanced in vitro and in vivo [39]. Here, we proved that the overexpression of WNT3a and CCND1 inhibited IHNV replication, probably via the enhancement of interferon response (Figure 6). It has been reported that the WNT signaling pathway and retinoic acid-inducible gene I (RIG-I)-like receptor (RLR)-dependent innate immune responses are linked during viral infection [40]. Therefore, the mechanisms by which IHNV interacts with the WNT pathway and how the WNT pathway regulates the IHNV-induced interferon response require further investigation.

## 5. Conclusions

In conclusion, this study demonstrated that IHNV upregulates the expression of miR-146a-3p at an early stage of the replication cycle. WNT3a and CCND1 were identified as the target genes of miR-146a-3p and were downregulated via miR-146a-3p during IHNV infection. Moreover, the miR-146a-3p-mediated enhancement of IHNV occurred in a WNT3a- and CCND1-dependent manner. WNT3a and CCND1 were positive regulators of IHNV replication through the inhibition of IFN response. Further study on the miR-146a-3p, WNT signaling pathway, and IHNV interactions might benefit the development of antiviral strategies.

## Figures and Tables

**Table 1:** Information of primers.

Gene Name	Primer	Sequences (5′ to 3′)	Application
NOTCH	NOTCH-1	AGGCCAACAGGTAAAACGATCAGAT	qRT-PCR
	NOTCH-2	CAGTGTCAGAACCCCACCTCACGAT	
DAAM	DAAM-1	ATCAGGAGTGACTCTCTAAGCTATT	
	DAAM-2	TCCGCCAGGGTCACTGCGTGTGT	
SOS	SOS-1	ATTCTCCCAGGTTTCTTCCCAAC	
	SOS-2	TGGTACACTGATATTGCCGTCAG	
CTBP	CTBP-1	GGCAAATCAGAGTTGGACAGGGA	
	CTBP-2	CTGTAATGGACCTCTGTGAGTCT	
WNT3a	WNT3a-1	ACAATCTTCTATCAATGCCTCGTCG	
	WNT3a-2	AACTCACTTTAGACTCTCTGACCAC	
CCND1	CCND1-1	ACGGACACCAAGAGCATGGATGA	
	CCND1-2	CCAGACCAGTCCCTGTGCTATGA	
IFN-I	IFN-I-1	AGAATGCCCCAGTCCTTTTCC	
	IFN-I-2	GACTTTGTCCTCAAACTCAGCATCA	
Mx	Mx-1	GGTTGTGCCATGCAACGTT	
	Mx-2	GGCTTGGTCAGGATGCCTAAT	
IHNV N	IHNV N-1	TGTGCATGAAGTCAGTGGTGG	
	IHNV N-2	CCTGCTCATCATGACACCGTA	
β-actin	β-actin-1	GCCGGCCGCGACCTCACAGACTAC	
	β-actin-2	CGGCCGTGGTGGTGAAGCTGTAAC	
miR-146a-3p	miR-146a-3p-1	TCGGCAGGATCTATGGGCTCAGT	
18S rRNA	18S rRNA-1	CGGAGGTTCGAAGACGATCA	
	18S rRNA-2	TCGCTAGTTGGCATCGTTTAT	
WNT3a-UTR	WNT3a-U1	CCGCTCGAGACAATCTTCTATCAATGCCTCGTCG	Reporter plasmid
	WNT3a-U2	GCGTCGACAACTCACTTTAGACTCTCTGACCAC	
WNT3a-MUT	WNT3a-mU1	TAGATACCCGAGTCAAGAAGACCACTTTCTTCAAAGAGCTTGCAG	
	WNT3a-mU2	GGTCTTCTTGACTCGGGTATCTACGATAGATGATAAAAAACAAAGT	
CCND1-UTR	CCND1-U1	CCGCTCGAGACGGACACCAAGAGCATGGATGA	
	CCND1-U2	GCGTCGACCCAGACCAGTCCCTGTGCTATGA	
CCND1-MUT	CCND1-mU1	TAGATACCCGAGTCAAGAAGACCTGCACCAGACAACACTGTCCCA	
	CCND1-mU2	GGTCTTCTTGACTCGGGTATCTAGGAGGAGAGGGCTTCGGGGAGA	
N	pN-1	*GGATCC*ACAAGCGCACTCAGAGAGA	Expression plasmid construction
	pN-2	*GAATTC*TCAGTGGAATGAGTCGGAGT	
P	pP-1	*GGTACC*TCAGATGGAGAAGGAGAAC	
	pP-2	*GAATTC*CTATTGACCCTGCTTCATGC	
M	pM-1	*GGATCC*TCCATTTTCAAGAGAGCA	
	pM-2	*GGAATTC*CTATTTTTCCTTCCCCCGT	
G	pG-1	*GGATCC*GACACCATGATCACCACT	
	pG-2	*GAATTC*TTAGGACCGGTTTGCCAG	
NV	pNV-1	*GGATCC*GACCACCGTGAAATAAAC	
	pNV-2	*GAATTC*CTATCTGGGATAAGCAAG	
L1	pL1-1	*GGTACC*GACTTCTTCGATCTCGACA	
	pL1-2	*GGATCC*CTACATGACGCGTTCTACCCT	
L2	pL2-1	*GGATCC*CAGAAAACAGCGCTCACCCA	
	pL2-2	*GAATTC*CTATTCCATGGGCATTGAGTA	
L3	pL3-1	*GGTACC*TCACAACGGCTCCTCCAC	
	pL3-2	*GAATTC*CTATGGTTCGCCTAGTGG	
WNT3a	pWNT3a-1	*GGATCC*GCCACCATGTTTTGCGAAACTTTTTT	
	pWNT3a-2	*CTCGAG*TCATTTGCAGGTGTGAACAT	
CCND1	pCCND1-1	*GGATCC*GCCACCATGGAACGTCAGTTGCTGTG	
	pCCND1-2	*CTCGAG*TCAGATGTTCACATCTCTCA	

Italic represents the restrictive endonuclease sites.

**Table 2:** The differentially expressed genes upon transfection with miR-146a-3p mimic.

Gene_ID	KEGG	KEGG_name	Fold_change	Pathway
110538410	K02599	NOTCH	0.81	Notch signaling pathway
110505746	K04512	DAAM	0.84	Wnt signaling pathway
110493792	K03099	SOS	0.88	MAPK signaling pathway
110504746	K04496	CTBP	0.90	Wnt signaling pathway
110530975	K00312	WNT3a	0.16	Wnt signaling pathway
110499952	K04503	CCND1	0.83	Wnt signaling pathway

## Data Availability

The data that support the findings of this study are available from the corresponding author, Cao, Y. S., upon reasonable request.

## Supplementary Materials

The following supporting information can be downloaded at: https://www.mdpi.com/article/10.3390/vetsci11050204/s1, Figure S1: Underlying data of Figure 4 Western Blot.
