# Supplementary material for: Host miR-146a-3p Facilitates Replication of Infectious Hematopoietic Necrosis Virus by Targeting WNT3a and CCND1
Source: Vet Sci. 2024 May 8;11(5):204. doi: 10.3390/vetsci11050204 (PMC11126136; doi:10.3390/vetsci11050204)
Supplement: Supplementary file 1 [file vetsci-11-00204-s001.zip › vetsci-2958280-supplementary.pdf]

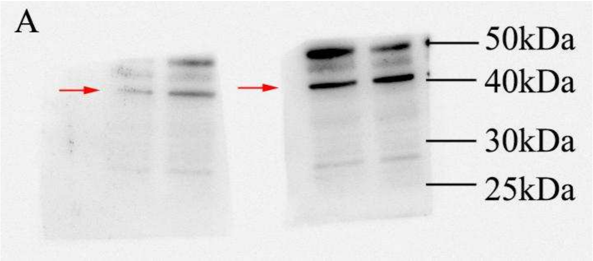
A
50kDa
40kDa
30kDa
25kDa

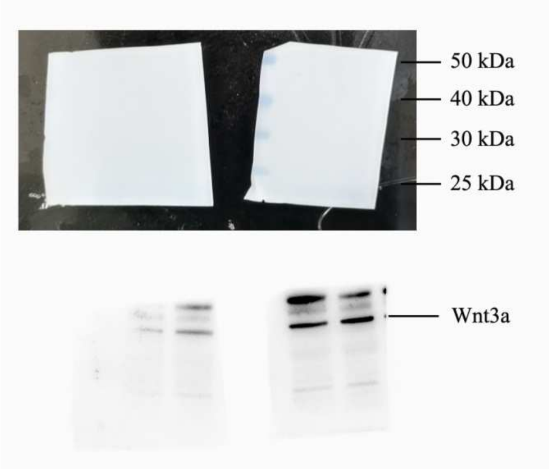
50 kDa
40 kDa
30 kDa
25 kDa
Wnt3a

A

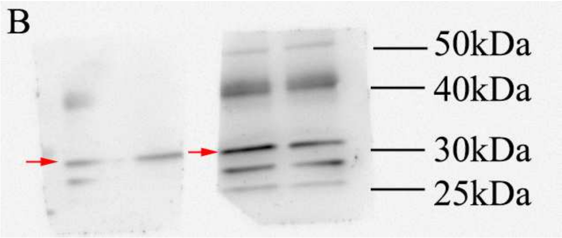
B
50kDa
40kDa
30kDa
25kDa

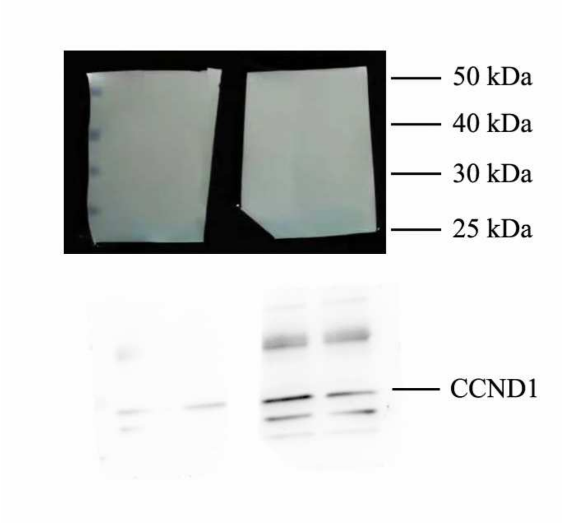
50 kDa
40 kDa
30 kDa
25 kDa
CCND1

B

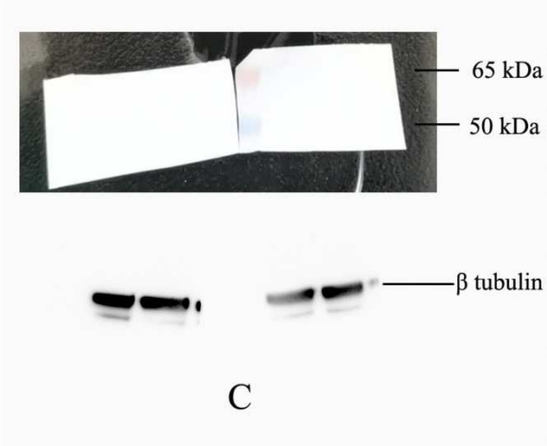

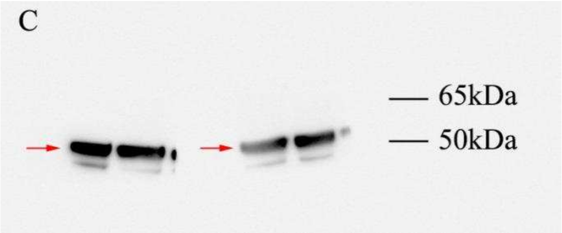

Raw data for the Western blotting analysis. RTG-2 cells were transfected with miRNA-146a-3p mimic or NC mimic and miRNA-146a-3p inhibitor or NC inhibitor for 24 h. Then, the cells were lysed and the target protein bands were detected using the corresponding antibody. The primary antibodies were anti-WNT3a (A), anti-CCND1(B), and anti-β tubulin (C).
